# First person – Amanda Havighorst

**DOI:** 10.1242/dmm.039255

**Published:** 2019-02-27

## Abstract

First Person is a series of interviews with the first authors of a selection of papers published in Disease Models & Mechanisms, helping early-career researchers promote themselves alongside their papers. Amanda Havighorst is first author on ‘Differential regulation of the unfolded protein response in outbred deer mice and susceptibility to metabolic disease’, published in DMM. Amanda is a PhD student in the lab of Dr Hippokratis Kiaris at the University of South Carolina, Columbia, USA, investigating individual variation in the unfolded protein response and how this variability affects susceptibility to metabolic disease.


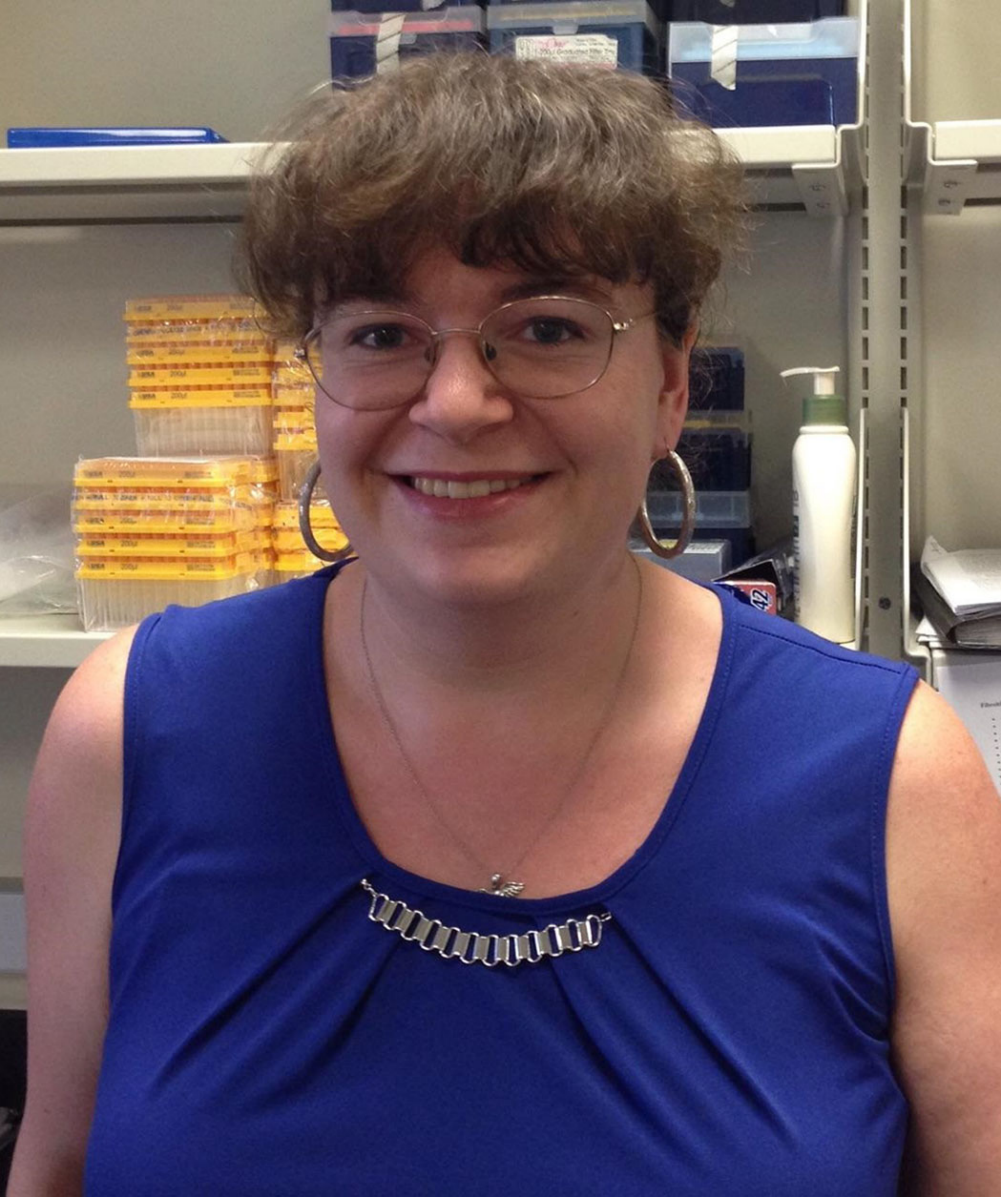


**Amanda Havighorst**

**How would you explain the main findings of your paper to non-scientific family and friends?**

Using cells collected from a genetically diverse group of rodents called deer mice, we demonstrated that the animals had individual differences in how strongly they responded to stress of the endoplasmic reticulum (ER), a compartment in the cells in which proteins are produced. Stress in the ER is associated with obesity-related conditions such as type II diabetes and fatty liver. By subjecting these animals to a diet designed to induce obesity, we were able to demonstrate a connection between the animals' innate ability to respond to ER stress and the likelihood of them developing obesity-related conditions, specifically fatty liver.

“[…] we have demonstrated a high variability of ER stress response in a genetically diverse population of rodents; it is probable that humans would display similar variability.”

**What are the potential implications of these results for your field of research?**

Obesity and metabolic disorders are becoming increasingly prevalent conditions in the developed world. However, the majority of studies being done on these conditions are performed on inbred laboratory mice, which do not reflect the genetic diversity seen in human populations. In this study, we have demonstrated a high variability of ER stress response in a genetically diverse population of rodents; it is probable that humans would display similar variability. Further, we have demonstrated that this variability affects the likelihood of developing metabolic diseases. This is significant within the field of personalized medicine; if we can test people in this manner, we could potentially predict their likelihood of developing certain conditions and educate them on preventative measures before the condition even develops.

**What are the main advantages and drawbacks of the model system you have used as it relates to the disease you are investigating?**

*Peromyscus maniculatus*, the deer mouse, is an outbred, genetically diverse model system, which more closely resembles the genetic diversity found in human populations than the standard laboratory mouse. This trait makes deer mice ideal for studies such as ours, which looks at how innate individual variation affects disease susceptibility. However, this same diversity means that they are not ideal for mechanistic studies; for such studies, the laboratory mouse is a better model due to its uniformity between individuals and the wide availability of experimental tools available for laboratory mouse research.

**What has surprised you the most while conducting your research?**

One of the most fascinating things I found was the degree of variability that exists between individuals. When I first began my studies, I did not expect that there would be such great variation in the levels of unfolded protein response among animals of the same species, and in their likelihood of developing obesity and fatty liver.

**Describe what you think is the most significant challenge impacting your research at this time and how will this be addressed over the next 10 years?**

Currently, there are very few experimental tools that can be used for deer mouse research. One can easily purchase, for example, antibodies for mice, rats and other common models, but no such resources exist for deer mice. I believe that, as the deer mouse gains traction as a model organism, these tools will become more readily available and allow for the exploration of new research questions that would more accurately be answered through the use of a genetically diverse model.

**Figure:**
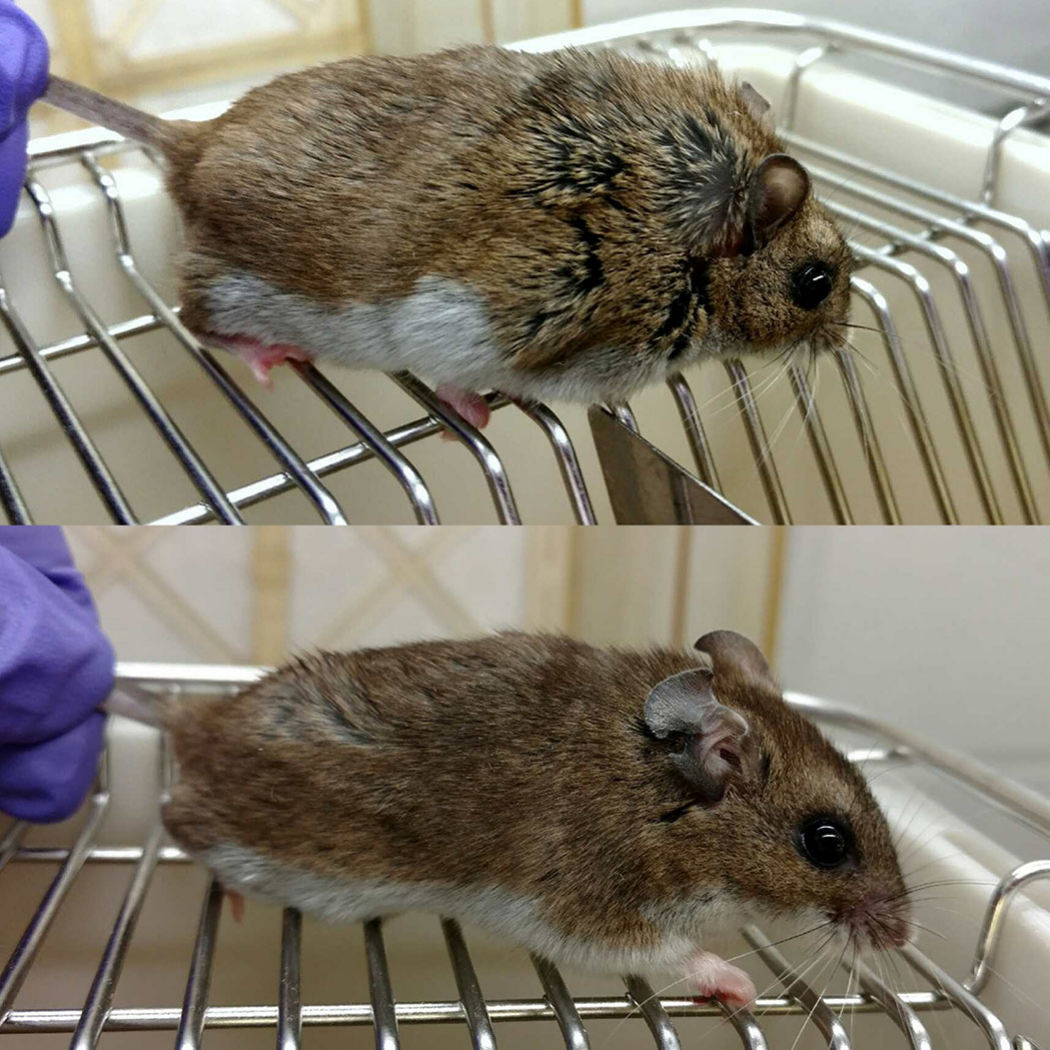
***Peromyscus* mice, fed the same diet for the same length of time, display differences in weight gain and – as observed later – development of steatosis, highlighting the genetic diversity of the animals and its importance in the development of disease.**

“One stumbling block that I believe many graduating PhD students face is the transition from being a student to entering the workforce.”

**What changes do you think could improve the professional lives of early-career scientists?**

One stumbling block that I believe many graduating PhD students face is the transition from being a student to entering the workforce. I believe that bridge funding during this transition, to support the graduating student as they prepare to begin their careers, would be a great boon to many of my peers and would allow them time to find an appropriate position that suits them well.

**What's next for you?**

My next objective is to perform *in vitro* studies to demonstrate how the severity of ER stress can affect the expression of genes related to lipogenesis.

## References

[DMM039255C1] Havighorst A., Zhang Y., Farmaki E., Kaza V., Chatzistamou I. and Kiaris H. (2019). Differential regulation of the unfolded protein response in outbred deer mice and susceptibility to metabolic disease. *Dis. Model. Mech.* 12, dmm037242 10.1242/dmm.037242 30733237 PMC6398494

